# Ambient fine particulate matter chemical composition associated with in-hospital case fatality, hospital expenses, and length of hospital stay among patients with heart failure in China

**DOI:** 10.7189/jogh.14.04032

**Published:** 2024-02-02

**Authors:** Zhihan Lai, Jingyi Zhang, Shanshan Ran, Dashan Zheng, Jin Feng, Gan Wu, Miao Cai, Hualiang Lin

**Affiliations:** Department of Epidemiology, School of Public Health, Sun Yat-sen University, Guangzhou, China

## Abstract

*Joint senior authorship.

**Background:**

Previous studies have observed the adverse effects of ambient fine particulate matter pollution (PM_2.5_) on heart failure (HF). However, evidence regarding the impacts of specific PM_2.5_ components remains scarce.

**Methods:**

We included 58 129 patients hospitalised for HF between 2013 and 2017 in 11 cities of Shanxi, China from inpatient discharge database. We evaluated exposure to PM_2.5_ and its components ((sulphate (SO_4_^2−^), nitrate (NO_3_^−^), ammonium (NH_4_^+^), organic matter (OM) and black carbon (BC)), along with meteorological factors using bilinear interpolation at each patients’ residential address. We used multivariable logistic and linear regression models to assess the associations of these components with in-hospital case fatality, hospital expenses, and length of hospital stay.

**Results:**

Increase equivalents to the interquartile range (IQR) in OM (odds ratio (OR) = 1.13; 95% confidence interval (CI) = 1.02, 1.26) and BC (OR = 1.14; 95% CI = 1.02, 1.26) were linked to in-hospital case fatality. Per IQR increments in PM_2.5_, SO_4_^2−^, NO_3_^−^, OM, and BC were associated with cost increases of 420.62 (95% CI = 285.75, 555.49), 221.83 (95% CI = 96.95, 346.71), 214.93 (95% CI = 68.66, 361.21), 300.06 (95% CI = 176.96, 423.16), and 303.09 (95% CI = 180.76, 425.42) CNY. Increases of 1 IQR in PM_2.5_, SO_4_^2−^, OM, and BC were associated with increases in length of hospital stay of 0.10 (95% CI = 0.02, 0.19), 0.09 (95% CI = 0.02, 0.17), 0.10 (95% CI = 0.03, 0.17), and 0.16 (95% CI = 0.08, 0.23) days.

**Conclusions:**

Our findings suggest that ambient SO_4_^2−^, OM, and BC might be significant risk factors for HF, emphasising the importance of formulating customised guidelines for the chemical constituents of PM and controlling the emissions of the most dangerous components.

Heart failure (HF), a clinical cardiac syndrome characterised by symptoms such as shortness of breath, fatigue, and fluid retention, has become as a prominent global public health concern and imposed substantial health burdens, including economic losses, disabilities, and fatalities [1,2]. It is the leading cause of mortality and morbidity in low- and middle-income countries [3]. As an ageing developing country, China is profoundly impacted by HF [4]; estimates suggest that approximately one-fifth of cardiovascular inpatients in China were affected by HF in 2003, and the related costs accounted for over 5% of the country’s total health care expenditure [5,6]. This identifying potential risk factors for HF and developing appropriate and effective preventive measures crucial for addressing this critical public health concern.

As a serious public health threat worldwide, air pollution has consistently been associated with elevated risks of cardiovascular diseases and related mortality [7–10]. Ambient particulate matter (PM) plays a vital role in this context; it constitutes a composite of solid and liquid particles, possessing aerodynamic diameters smaller than 2.5 µm (PM_2.5_) and ranging between 2.5 and 10 µm (PM_10–2.5_) [11]. According to the updated 2021 PM_2.5_ thresholds of the World Health Organization (WHO), approximately 1.41 billion people in China (99% of the total population) were exposed to unsafe levels of PM_2.5_ (>5 μg/m^3^), while 765 million (53% of the population) resided in areas with hazardous air quality (>35 μg/m^3^) [12]. Numerous studies found favourable associations between ambient PM and increased incidence, hospitalisation, readmission, and mortality of HF [13–16]. However, they primarily used incidence or mortality rates of HF to assess the disease burden attributed to air pollution, without emphasising metrics such as hospitalisation expenses and length of hospital stay (LOS). These indicators better describe the influence of air pollution on individuals and give additional practical significance. Additionally, most studies used the mass concentration of PM as the indicator, overlooking the toxicity associated with its different chemical components ((sulphate (SO_4_^2−^), nitrate (NO_3_^−^), ammonium (NH_4_^+^), organic matter (OM), and black carbon (BC)). Their health effects are unlikely to be equally significant, considering the distinct chemical properties of each PM chemical component. Though WHO released the latest air quality guidelines in 2021, specific guidelines for these chemical components are still unavailable. Previous studies have shown that they can impair human health through different mechanisms such as systemic inflammation, oxidative stress, adipose tissue inflammation, impaired vascular function, and insulin resistance [17,18]. Therefore, studying the relationships between PM chemical components and HF could help with advancing the current understanding of PM toxicity and facilitating the development of more focused regulations and policies aimed at reducing the health burden caused by PM air pollution.

We hypothesised that PM_2.5_ and its chemical constituents are associated with increased risks of in-hospital mortality, hospitalisation costs, and LOS in patients hospitalised due to HF. We explored this by using data from the inpatient hospitalisation database of Shanxi, a province known for its highly developed coal mining industry and poor air quality in China. We assessed the impact of individual-level exposure to PM_2.5_ mass and its dust-free chemical constituents at HF patients’ residential addresses prior to hospitalisation.

## METHODS

### Study area

Shanxi Province, located on the eastern boundary of the Loess Plateau and west of Taihang Mountains in the northern region of China, stretches over 156 700 km^2^. It administratively encompasses 11 prefecture-level cities (Changzhi, Datong, Jincheng, Jinzhong, Linfen, Lvliang, Shuozhou, Taiyuan, Xinzhou, Yangquan and Yuncheng) with a population of 34.9 million in 2020. Due to the temperate continental climate, Shanxi is relatively dry and undergoes large temperature differences all year round. As a province rich in coal resources, Shanxi Province has a highly developed coal-related industry, which has resulted in severe air pollution problems, significantly impacting the health of residents.

### Data collection

We retrieved data on individual patient’s demographics, medical diagnoses and procedures, hospital costs, days of hospital stay, residential addresses, and in-hospital health outcomes from the electronic medical records system of secondary and tertiary hospitals of Shanxi Province [19–22]. The data spanned a period from 13 February 2013 to 31 December 2017. Before accessing the data, personal identifiers such as names and distinct identification codes of patients were removed. This study received ethical approval from the institutional review board of the School of Public Health, Sun Yat-sen University.

### Identification of HF

**Figure 1:**
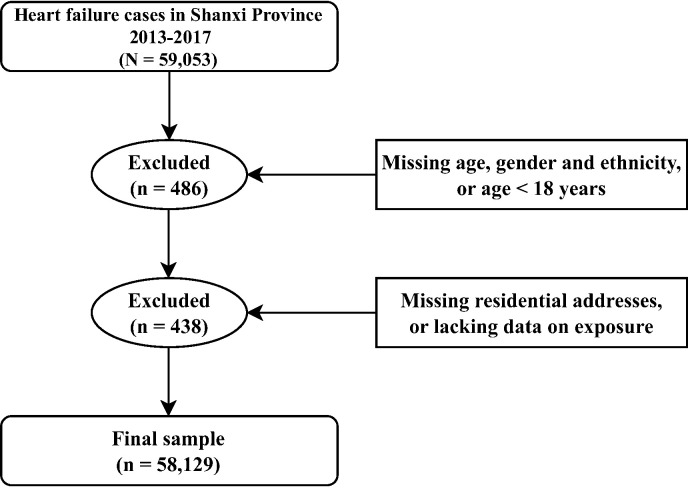
Flowchart of sample selection, inclusion and exclusion criteria.

We used the I50 primary diagnosis code at admission (per the International Classification of Diseases, 10th Edition (ICD-10)) to identify HF cases. We excluded individuals <18 years of age, those with unknown age, sex, ethnicity, or residential addresses, and those lacking data on exposure (**Figure 1**).

### Air pollution exposure measurement

We obtained daily measurements of PM_2.5_ mass and its dust-free chemical constituents (SO_4_^2−^, NO_3_^−^, NH_4_^+^, OM, and BC) with a spatial resolution of 10 × 10 km from the Tracking Air Pollution database in China [23–25]. The database integrates ground-based measurements, satellite-derived aerosol optical depth estimations, and emissions data from inventories to calculate the daily concentrations of PM chemical components. These estimates are generated using the Weather Research and Forecasting (version 3.9.1)-Community Multiscale Air Quality (version 5.2) chemical transport models in China since 2000 until the current date [23]. Providing information of daily concentrations of PM chemical components, the Tracking Air Pollution data set shows a mean out-of-bag cross-validation correlation coefficient (R) of 0.83. The cross-validation R values for the model were 0.70 for SO_4_^2−^, 0.75 for NO_3_^−^, 0.75 for NH_4_^+^, 0.72 for OM and 0.64 for BC, respectively, indicating strong agreement with ground measurements of PM chemical constituents [23,24].

We employed a two-step assessment strategy to estimate the average individual-level exposure to PM_2.5_ mass and its constituent chemicals, along with temperature and relative humidity, over a period of seven days. The first step involves geocoding the latitude and longitude coordinates of the study patients through the application programming interface (API) provided by ‘amap’ (Gaode map) based on their residential addresses prior to hospitalisation. In the second step, bilinear interpolation is used for the purpose of assessing the magnitudes of environmental variables, including PM_2.5_ mass and its chemical components, temperature, and relative humidity, at the residential addresses of the patients prior to their hospitalisation. The detailed calculation process can be found elsewhere [26,27]. Briefly, this algorithm improved the spatial precision of environmental variables at particular locations through the calculation of a weighted average derived from the closest four grid points, and the weights were determined by the distance between the case address and the grids of meteorologic variables, with closer distances having higher weights [28,29].

Short-term exposure was characterised by the seven-day average concentrations of PM_2.5_ and its constituents, along with the temperature and relative humidity leading up to the day of hospitalisation.

### Outcomes

We obtained our outcomes of interest (in-hospital case fatality, hospitalisation expenses (CNY) and length of hospital stay (LOS in days)) from the collected electronic medical records. The hospital expenses encompass the subsequent elements: charges for medications, clinical examinations, nursing expenditures, and clinical operations. LOS refers to the cumulative number of days a patient remains hospitalised from admission to discharge.

### Covariates

We selected the following covariates based on their availability in the database and their potential to confound the associations between PM_2.5_ mass, its chemical constituents, and HF: demographic variables, socioeconomic status, comorbidities, hospital level, payment methods and meteorological status. The demographic variables comprised age, sex, and ethnicity (Han vs non-Han). Socioeconomic status encompassed occupation (public sector, private sector, agriculture, unemployed, retired, or other) and marital status (married, single, widowed, divorced, or other). We determined existing comorbidities (Charlson comorbidity index) by extracting ICD-10 codes from the patient diagnosis fields, which we refined through text matching using regular expressions applied to clinical diagnosis descriptions [30]. We categorised hospital level into either ‘tertiary’ or ‘non-tertiary’ [19], and tertiary hospital represents a higher level of medical care. Payment methods included the New Rural Cooperative Medical Scheme, Urban Employee Basic Medical Insurance, Urban Resident Basic Medical Insurance, self-payment, and others. We collected daily meteorological information on temperature and relative humidity with a spatial resolution of 9 × 9 km through the fifth generation of European ReAnalysis– Land reanalysis data set [31,32]. We employed natural cubic splines with five degrees of freedom to incorporate the seven-day average temperature and relative humidity leading up to the day of hospitalisation. In this way, we aimed to accommodate potential nonlinear associations, considering that earlier studies have suggested the presence of relationships displaying J-shaped or U-shaped patterns. [33].

### Statistical analyses

We summarised the sample chracteristics, stratified by fatality status, using means and standard deviations (SDs) for continuous and frequency with percentages for categorical variables. We used Spearman’s rank correlation to estimate the correlations between each pair of PM_2.5_ and its components.

We employed logistic regression models to estimate the odds ratios (ORs) of in-hospital case mortality for each interquartile range (IQR) increase in PM_2.5_ and its chemical components, along with 95% confidence intervals (CIs). Hospitalisation expenses and LOS showed a positively skewed distribution with pronounced tails. Nevertheless, considering the significant size of our study sample and considering the distribution patterns (Figure S1–2 in the **Online Supplementary Document**), we opted to use linear regression to investigate the association between exposure and these variables to more clearly describe influence of exposure on hospitalisation outcomes in patients with HF. We also used nonlinear categorical variable (quartile modes) of PM_2.5_ and its chemical components to repeat the above analyses and conducted trend tests. Additionally, to demonstrate potential nonlinear associations and estimate exposure-response curves, we incorporated PM_2.5_ and its chemical components as natural cubic splines with knots specified at quartiles. We did not use multi-pollutant models, since most air pollutants had moderate to high correlations with one another (Table S1 in the **Online Supplementary Document**).

We performed sensitivity analyses to assess the robustness of the findings. First, we evaluated various time lags (lag 0 to lag 7) of short-term exposure to PM_2.5_ and its chemical components using bilinear interpolation. Second, we only included participants with a main diagnosis of HF (n = 44 565, 76.7% of the full sample) in this study to repeat our analyses. Third, we excluded the uppermost 1% of data points for hospitalisation expenses (>47 652.27 CNY, n = 582) and LOS (>29 days, n = 647) to mitigate the influence of extreme values from these two right-skewed distributed variables. Lastly, we employed the interval between admission date and discharge date as the follow-up time and constructed Cox regression models to examine the associations between these pollutants and in-hospital mortality among HF patients.

All data processing, including data cleaning, statistical modelling, and data visualisation, were executed in R, version 4.2.1 (R Core Team, Vienna, Austria). We considered a two-way *P*-value <0.05 as statistically significant.

## RESULTS

### Descriptive results

The 58 129 patients with a primary diagnosis of HF had a mean age of 69.21 years, and 48.0% were female (**Table 1**). Among them, 630 (1.08%) experienced hospital fatality; they were more likely to be retired, divorced or widowed, receive treatment at tertiary hospitals, utilise Urban Resident Basic Medical Insurance, experience comorbidities, and have higher hospitalisation costs and shorter LOS. The seven-day average concentrations prior to hospitalisation were 62.14 (SD = 27.76) μg/m^3^ for PM_2.5_, 10.40 (SD = 4.05) μg/m^3^ for SO_4_^2−^, 12.52 (SD = 7.39) μg/m^3^ for NO_3_^−^, 8.24 (SD = 4.23) μg/m^3^ for NH_4_^+^, 14.64 (SD = 7.59) μg/m^3^ for OM and 2.87 (SD = 1.31) μg/m^3^ for BC. The mean concentrations of PM_2.5_, SO_4_^2−^, OM, and BC were higher in those who experienced hospital fatality, though the difference for SO_4_^2−^ was not statistically significant.

**Table 1:** Environmental variables and patient characteristics by case fatality

		Case fatality		
	**Overall (n = 58 129)**	**No (n = 57 499)**	**Yes (n = 630)**	***P*-value**
**Seven-day average concentrations prior to hospitalisation, μg/m^3^, x̄ (SD)**				
PM_2.5_	62.14 (27.76)	62.10 (27.73)	65.89 (30.11)	0.001
SO_4_^2−^	10.40 (4.05)	10.40 (4.05)	10.55 (4.22)	0.36
NO_3_^−^	12.52 (7.39)	12.52 (7.39)	12.35 (7.32)	0.558
NH_4_^+^	8.24 (4.23)	8.24 (4.23)	7.99 (4.13)	0.142
OM	14.64 (7.59)	14.63 (7.57)	15.90 (8.64)	<0.001
BC	2.87 (1.31)	2.87 (1.30)	3.12 (1.50)	<0.001
**Demographics and socioeconomic status**				
Age in years, x̄ (SD)	69.21 (34.18)	69.19 (34.31)	71.16 (18.28)	0.151
Sex, n (%)				0.083
*Female*	27 877 (48.0)	27 597 (48.0)	280 (44.4)	
*Male*	30 252 (52.0)	29 902 (52.0)	350 (55.6)	
Ethnicity, n (%)				0.517
*Han*	56 742 (97.6)	56 130 (97.6)	612 (97.1)	
*Non-Han*	1387 (2.4)	1369 (2.4)	18 (2.9)	
Occupation, n (%)				<0.001
*Farmer*	29 672 (51.0)	29 489 (51.3)	183 (29.0)	
*Jobless*	3119 (5.4)	3096 (5.4)	23 (3.7)	
*Other*	5791 (10.0)	5707 (9.9)	84 (13.3)	
*Private institution*	6534 (11.2)	6476 (11.3)	58 (9.2)	
*Public institution*	1443 (2.5)	1433 (2.5)	10 (1.6)	
*Retired*	11 570 (19.9)	11 298 (19.6)	272 (43.2)	
Marriage, n (%)				<0.001
*Divorced*	926 (1.6)	898 (1.6)	28 (4.4)	
*Married*	50 419 (86.7)	49 931 (86.8)	488 (77.5)	
*Other*	1470 (2.5)	1451 (2.5)	19 (3.0)	
*Unmarried*	1620 (2.8)	1593 (2.8)	27 (4.3)	
*Widowed*	3694 (6.4)	3626 (6.3)	68 (10.8)	
**Comorbidities**				
Hypertension, n (%)	29 234 (50.3)	28 981 (50.4)	253 (40.2)	<0.001
Diabetes, n (%)	10 380 (17.9)	10 240 (17.8)	140 (22.2)	0.005
Cardiac arrhythmias, n (%)	25 370 (43.6)	25 093 (43.6)	277 (44.0)	0.901
Peripheral vascular disorders, n (%)	5280 (9.1)	5258 (9.1)	22 (3.5)	<0.001
Liver disease, n (%)	3666 (6.3)	3638 (6.3)	28 (4.4)	0.064
Charlson comorbidity index, x̄ (SD)	4.71 (1.68)	4.71 (1.67)	5.08 (1.95)	<0.001
**Medical status**				
Hospital level, n (%)				<0.001
*Non-tertiary*	27 455 (47.2)	27 240 (47.4)	215 (34.1)	
*Tertiary*	30 674 (52.8)	30 259 (52.6)	415 (65.9)	
Payment method, n (%)				<0.001
*NRCMS*	30 301 (52.1)	30 127 (52.4)	174 (27.6)	
*Other*	2742 (4.7)	2690 (4.7)	52 (8.3)	
*Self-payment*	4031 (6.9)	3976 (6.9)	55 (8.7)	
*UEBMI*	16 690 (28.7)	16 401 (28.5)	289 (45.9)	
*URBMI*	4365 (7.5)	4305 (7.5)	60 (9.5)	
Total cost in CNY, x̄ (SD)	8976.14 (10 758.68)	8899.73 (10 407.50)	15950.56 (27316.17)	<0.001
LOS in days, x̄ (SD)	9.90 (6.28)	9.92 (6.11)	8.23 (15.18)	<0.001
**Meteorologic variables (seven-day average prior to hospitalisation), x̄ (SD)**				
Temperature in °C	10.16 (9.75)	10.17 (9.75)	9.21 (10.06)	0.014
Relative humidity in %	53.17 (14.79)	53.18 (14.78)	52.08 (15.17)	0.063

BC – black carbon, NH_4_^+^ – ammonium, NO_3_^−^ – nitrate, NRCMS – New Rural Cooperative Medical Scheme, OM – organic matter, PM_2.5_ – particulate matter <2.5 μm in aerodynamic diameter, SD – standard deviation, SO_4_^2−^ – sulphate, UEBMI – Urban Employee Basic Medical Insurance, URBMI – Urban Resident Basic Medical Insurance, x̄ – mean

### Associations of PM_2.5_ and its chemical components with in-hospital case fatality, hospitalisation expenses, and LOS

We further explored associations of PM_2.5_ and its chemical components as continuous variables with in-hospital case fatality, hospitalisation expenses, and LOS (**Table 2**). Among the three indicators, OM and BC both showed statistically significant associations. For each increase in one IQR, OM was associated with a 13% rise in the risk of in-hospital fatality (OR = 1.13; 95% CI = 1.02, 1.26), accompanied by individual cost and LOS increases of 300.06 CNY (95% CI = 176.96, 423.16) and 0.10 days (95% CI = 0.03, 0.17), respectively. BC corresponded to a 14% increase in the risk of in-hospital fatality (OR = 1.14, 95% CI = 1.02, 1.26), with individual cost and LOS increases of 303.09 CNY (95% CI = 180.76, 425.42) and 0.16 days (95% CI = 0.08, 0.23), respectively. Regarding other pollutants, PM_2.5_ and SO_4_^2−^ were associated with increased costs and LOS, while NO_3_^-^ is only related to increased costs. We found no statistically significant associations with NH_4_^+^.

**Table 2:** Associations of PM_2.5_ and its chemical components (each IQR increment) with in-hospital case fatality, hospitalisation cost, and LOS*

Air pollutant	IQR in μg/m^3^	Fatality, OR (95% CI)	Cost in CNY, β (95% CI)	LOS in days, β (95% CI)
PM_2.5_	33.16	1.10 (0.97, 1.23)	420.62 (285.75, 555.49)	0.10 (0.02, 0.19)
SO_4_^2−^	5.13	1.01 (0.90, 1.13)	221.83 (96.95, 346.71)	0.09 (0.02, 0.17)
NO_3_^−^	9.07	0.90 (0.79, 1.04)	214.93 (68.66, 361.21)	0.02 (−0.06, 0.11)
NH_4_^+^	5.17	0.88 (0.77, 1.00)	128.63 (−6.37, 263.63)	0.02 (−0.06, 0.10)
OM	8.43	1.13 (1.02, 1.26)	300.06 (176.96, 423.16)	0.10 (0.03, 0.17)
BC	1.51	1.14 (1.02, 1.26)	303.09 (180.76, 425.42)	0.16 (0.08, 0.23)

β – correlation coefficient, BC – black carbon, CI – confidence interval, IQR – interquartile range, NH_4_^+^ – ammonium, NO_3_^−^ – nitrate, OM – organic matter, OR – odds ratio, PM_2.5_ – particulate matter <2.5 μm in aerodynamic diameter, SO_4_^2−^ – sulphate

*Adjusted for age, sex, ethnicity, occupation, marital status, payment methods, comorbidities, hospital level, temperature and humidity.

When using air pollutants as categorical variables categorized from lower concentration (quartile 1) to higher concentration (quartile 4), no statistically significant association with in-hospital case fatality could be found (**Table 3**). Regarding hospitalisation cost, the trend tests for all pollutants showed statistical significance (*P*-value for trend <0.05). Regarding the LOS, only PM_2.5_, OM and BC showed statistical significance in the trend tests.

**Table 3:** Associations of PM_2.5_ and its chemical components with in-hospital case fatality, hospitalisation cost, and LOS, using air pollution as categorical variables*

	Fatality		Cost in CNY		LOS in days	
**Air pollutants by quartiles**	**OR (95% CI)**	***P*-value for trend**	**β (95% CI)**	***P*-value for trend**	**β (95% CI)**	***P*-value for trend**
PM_2.5_		0.971		<0.001		0.019
*1*	ref		ref		ref	
*2*	0.86 (0.68, 1.09)		602.13 (360.95, 843.32)		−0.08 (−0.23, 0.06)	
*3*	0.87 (0.68, 1.12)		752.67 (494.53, 1010.81)		0.12 (−0.03, 0.28)	
*4*	1.00 (0.76, 1.33)		1022.95 (716.83, 1329.07)		0.18 (0.00, 0.36)	
SO_4_^2−^		0.720		<0.001		0.144
*1*	ref		ref		ref	
*2*	0.85 (0.67, 1.07)		372.10 (124.92, 619.28)		0.02 (−0.13, 0.17)	
*3*	0.83 (0.65, 1.05)		668.30 (413.30, 923.30)		0.04 (−0.12, 0.19)	
*4*	0.95 (0.74, 1.22)		566.29 (294.20, 838.39)		0.12 (-0.04, 0.29)	
NO_3_^−^		0.163		<0.001		0.613
*1*	ref		ref		ref	
*2*	0.70 (0.55, 0.89)		470.43 (222.42, 718.44)		0.02 (−0.13, 0.17)	
*3*	0.76 (0.59, 0.99)		552.64 (277.01, 828.28)		−0.11 (−0.28, 0.05)	
*4*	0.79 (0.58, 1.06)		624.74 (294.44, 955.05)		0.00 (−0.20, 0.20)	
NH_4_^+^		0.132		0.024		0.550
*1*	ref		ref		ref	
*2*	0.76 (0.60, 0.95)		292.72 (44.89, 540.55)		0.01 (−0.14, 0.16)	
*3*	0.78 (0.61, 0.99)		359.62 (95.68, 623.57)		−0.09 (−0.25, 0.07)	
*4*	0.80 (0.60, 1.05)		346.67 (42.20, 651.14)		−0.02 (−0.20, 0.17)	
OM		0.762		<0.001		<0.001
*1*	ref		ref		ref	
*2*	0.82 (0.65, 1.05)		630.95 (389.42, 872.48)		0.02 (−0.13, 0.16)	
*3*	0.92 (0.72, 1.17)		522.42 (267.73, 777.10)		0.16 (0.02, 0.33)	
*4*	1.04 (0.79, 1.37)		918.09 (615.24, 1220.94)		0.18 (0.12, 0.48)	
BC		0.618		<0.001		<0.001
*1*	ref		ref		ref	
*2*	1.03 (0.81, 1.30)		487.50 (245.26, 729.94)		0.14 (0.00, 0.29)	
*3*	0.90 (0.70, 1.15)		467.80 (216.66, 718.93)		0.26 (0.11, 0.41)	
*4*	1.13 (0.86, 1.47)		778.93 (493.01, 1064.85)		0.38 (0.21, 0.55)	

β – correlation coefficient, BC – black carbon, CI – confidence interval, IQR – interquartile range, LOS – length of hospital stay, NH_4_^+^ – ammonium, NO_3_^−^ – nitrate, OM – organic matter, OR – odds ratio, PM_2.5_ – particulate matter <2.5 μm in aerodynamic diameter, ref – reference, SO_4_^2−^ – sulphate

*Adjusted for age, sex, ethnicity, occupation, marital status, payment methods, comorbidities, hospital level, temperature and humidity.

**Figure 2:**
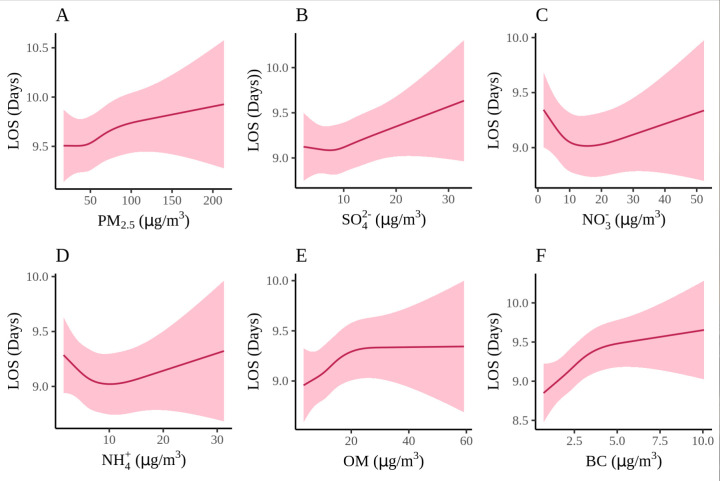
Exposure-response relationships of seven-day average PM_2.5_ and its chemical compositions with HF LOS. **Panel A.** Particulate matter <2.5 μm in aerodynamic diameter (PM_2.5_). **Panel B.** Sulphate (SO_4_^2−^). **Panel C.** Nitrate (NO_3_^−^). **Panel D.** Ammonium (NH_4_^+^). **Panel E.** Organic matter (OM). **Panel F.** Black carbon (BC).

For hospitalisation cost, PM_2.5_, OM, and BC displayed a consistent positive association with higher costs as their concentrations increased. Notably, curves of these three pollutants demonstrated a rapid escalation in cost at lower concentrations, followed by a more moderate increase at higher concentrations, while curves of SO_4_^2−^, NO_3_^−^ and NH_4_^+^ demonstrated a distinct pattern, with rapid increase at lower concentrations but a slight decrease at higher concentrations. As for LOS, the exposure-response relationships of PM_2.5_, OM, and BC demonstrated an overall increasing trend, while SO_4_^2−^, NO_3_^−^ and NH_4_^+^ exhibited a decreasing trend at low concentrations, followed by an increase at high concentrations (**Figure 2**; Figure S3 in the **Online Supplementary Document**).

### Sensitivity analyses

The results of various time lags (lag 0 to lag 7) of short-term exposure to PM_2.5_ and its chemical components demonstrated considerable robustness. Meanwhile, our findings changed minimally for participants only with a main diagnosis of HF and remained largely consistent after excluding the top 1% of extreme values of hospitalisation expenses and LOS. The Cox regression analysis likewise showed consistent (Figures S4–6 and Tables S3–5 in the **Online Supplementary Document**).

## DISCUSSION

Based on a sample of 58 129 HF patients from Shanxi, a region known for severe air pollution, we studied the association of PM_2.5_ and its chemical components with in-hospital case fatality, hospitalisation cost, and LOS. We found a positive association between the concentrations of two PM_2.5_ components (OM and BC) and in-hospital fatality of HF patients. Our results also indicated that higher concentrations of PM_2.5_ and its components (SO_4_^2−^, NO_3_^−^, OM, and BC) were associated with increased hospitalisation cost, while elevated concentrations of PM_2.5_, SO_4_^2−^, OM, and BC were associated with longer LOS. We also conducted several sensitivity analyses to demonstrate the robustness of the results, where our findings regarding OM and BC showed the highest level of significance and consistency, showing statistical significance in all three indicators.

Several previous studies have explored the associations between these components and cardiovascular health. The National Particle Component Toxicity report indicated that the increased incidence of cardiovascular diseases is associated with exposure to OM [34]. Prior studies reported on the harmful impact of OM on cardiopulmonary mortality and overall mortality [35,36]. We also identified the adverse effects of OM on HF patients. However, this is inconsistent with some previous studies which no association between OM and the incidence of CVDs [37]. This difference might be due to variations in geographic regions, air pollution exposure patterns, and population characteristics, and thus warrants further exploration. The association between BC and CVDs appear to be more substantial, with several studies reporting the adverse effects of BC on cardiovascular health [37–40], which aligns with our findings.

There is limited evidence on the impact of secondary inorganic ions, including SO_4_^2−^, NO_3_^−^ and NH_4_^+^, on cardiovascular health. A previous cohort study found that these three ions have long-term adverse effects on the incidence of CVDs [37]. However, in our study, NO_3_^−^ was solely linked to increased hospitalisation cost in HF patients, aligning with prior research indicating minimal or negligible adverse health effects of nitrate at current levels, while toxicological studies utilised nitrate concentrations significantly exceeding environmental levels [41]. Furthermore, we observed no significant association between NH_4_^+^ and HF patients. A recent meta-analysis suggested that more in-depth investigations are still required to elucidate the impact of these ions as air pollutants on health [42]. Notably, exposure-response relationship curves indicate that SO_4_^2−^, NO_3_^−^ and NH_4_^+^ exhibit a U-shaped curve with a slight initial decrease followed by an increase for LOS, yet demonstrate an inverted U-shaped curve for hospitalisation costs. This suggests that these dose-response relationships are likely nonlinear, necessitating external data for a more comprehensive understanding.

These observed associations could be explained by several plausible biological mechanisms. Previous studies in both animals and humans have indicated that PM may damage the cardiovascular system through oxidative stress, systemic inflammation, alterations in cardiac autonomic function, and acceleration of atherosclerosis [43–45]. For example, BC has been linked to adverse cardiovascular outcomes, potentially mediated by its ability to induce systemic inflammation and oxidative stress through down-regulating DNA methylation [46]. Upon penetration of lung tissues and entry into the bloodstream, BC, OM, and SO_4_^2−^ can trigger various physiological processes, including oxidative stress, vascular inflammation responses, and coagulation and thrombosis mechanisms [47,48]. Notably, longer-term exposure to PM over several years posed a greater risk for cardiovascular mortality compared to shorter-term exposures lasting only a few days [49], which indicated that the adverse effects of PM observed in this study may still be underestimated.

The sources of PM_2.5_ components have been described elsewhere [26,40,50]. SO_4_^2−^ mainly comes from human fossil fuel combustion and natural sources, including sulphur-containing gases emitted from the ocean and volcanoes. OM is primarily generated from a combination of emissions from combustion sources and secondary reactions, including biogenic volatile organic compounds, and BC is solely produced from incomplete combustion in various sources such as vehicular traffic, industrial emissions and residential biofuel burning. The detrimental impact of BC and OM on human health is exacerbated by their contribution to intensifying heat-absorbing greenhouse effects and contributing to global warming, akin to carbon dioxide [51,52]. This additional impact on human health emphasises the significance of addressing these pollutants in environmental regulations and public health interventions.

Our study has several major strengths. To the best of our knowledge, it is the first one conducted in China to explore the relationship between ambient fine particulate matter chemical components and HF, focusing on in-hospital mortality, hospitalisation cost, and LOS in HF patients, and thus assesses the health burden of air pollution exposure on HF from multiple perspectives. Our results can provide preliminary evidence for policymakers and regulatory authorities to establish threshold levels for these components and to implement control measures for emission sources. Compared with developed regions in the West (Europe and North America), China experiences more severe air pollution exposure, making it conducive to studying the toxicity of different ambient fine particulate matter chemical components. Moreover, the exposure assessment in this study was conducted at the individual level with high temporal (daily) and spatial (10km ×10km grid) resolution, resulting in greater accuracy in measurements.

However, few limitations should be noted. As this is a cross-sectional study, we could not establish a causal relationship between exposure and outcomes. We also only included HF patients from secondary and tertiary hospitals, which inevitably introduced selection bias. The findings were based on data from Shanxi Province, which limited the applicability to other provinces and emerging nations. We should also acknowledge that certain potential confounders, such as cigarette smoking, alcohol consumption, and physical activity, could not be completely ruled out in our study due to data constraints. However, the inclusion of surrogate variables (such as age, sex, and occupation) in the regression models might have helped mitigate potential biases. Likewise, we primarily focused on hospitalised HF patients who may also have various other coexisting conditions, some of which might similarly be affected by air pollutants. However, we could not provide explanations for the relationship between PM_2.5_ and its chemical components and these additional diseases due to the study design. The Tracking Air Pollution data set primarily focused on monitoring outdoor concentrations of air pollutants, so we could not assess the concentrations of indoor PM_2.5_ chemical components. Finally, the high degree of so between components and the absence of suitable multi-component statistical models limited our ability to explore potential interactions among various PM_2.5_ chemical components.

## CONCLUSIONS

We found that elevated concentrations of PM_2.5_ chemical components, particularly OM and BC, are associated with increased hospitalisation mortality, costs, and LOS among HF patients. It is crucial to establish guidelines for PM_2.5_ chemical components and regulate the emissions of the most perilous constituents to enhance the health condition of this population.

## Additional material


Online Supplementary Document
