# Supplementary material for: Ambient fine particulate matter chemical composition associated with in-hospital case fatality, hospital expenses, and length of hospital stay among patients with heart failure in China
Source: J Glob Health. 2024 Feb 2;14:04032. doi: 10.7189/jogh.14.04032 (PMC10832573; doi:10.7189/jogh.14.04032)
Supplement: Online Supplementary Document [file jogh-14-04032-s001.pdf]

Supplementary materials

Table S1 Correlation coefficients between different pollutants

	$PM_{2.5}$	SO_4^{2-}	NO_3^-	NH_4^+	OM	BC
$PM_{2.5}$	1.00					
SO_4^{2-}	0.89	1.00				
NO_3^-	0.93	0.86	1.00			
NH_4^+	0.91	0.91	0.98	1.00		
OM	0.96	0.80	0.82	0.80	1.00	
BC	0.92	0.80	0.76	0.75	0.97	1.00

Tables S2 Associations of $PM_{2.5}$ and its chemical components with in-hospital case fatality, hospitalization expenses and LOS, results of participants only with a main diagnosis of HF

Air pollutant	IQR (μg/m^3)	Hospital death	Cost (RMB)	LOS (days)
		OR (95% CI)	β (95% CI)	β (95% CI)
$PM_{2.5}$	33.02	1.09 (0.94, 1.25)	429.80 (283.56, 576.04)	0.11 (0.01, 0.19)
SO_4^{2-}	5.14	1.00 (0.87, 1.15)	232.51 (96.63, 368.40)	0.10 (0.01, 0.19)
NO_3^-	9.07	0.83 (0.70, 0.98)	160.66 (2.12, 319.21)	0.03 (-0.07, 0.13)
NH_4^+	5.20	0.81 (0.69, 0.94)	96.06 (-52.71, 240.82)	0.02 (-0.07, 0.12)
OM	8.32	1.16 (1.02, 1.31)	314.53 (180.04, 449.03)	0.09 (0.00, 0.17)
BC	1.48	1.16 (1.02, 1.32)	324.99 (192.00, 457.99)	0.15 (0.06, 0.23)

Table S3 Associations of $PM_{2.5}$ and its chemical components with hospitalization expenses, excluding the top 1% of extreme values

Air pollutant	IQR (μg/m^3)	β（95% CI）	P
$PM_{2.5}$	33.13	374.34 (298.22, 450.46)	<0.001
SO_4^{2-}	5.13	219.64 (149.09, 290.20)	<0.001
NO_3^-	9.07	168.95 (86.26, 251.63)	<0.001
NH_4^+	5.17	107.93 (31.62, 184.23)	0.006
OM	8.43	286.33 (216.82, 355.84)	<0.001
BC	1.51	293.20 (224.12, 362.28)	<0.001

Table S4 Associations of $PM_{2.5}$ and its chemical components with LOS, excluding the top 1% of extreme values

Air pollutant	IQR (μg/m^3)	β（95% CI）	P
$PM_{2.5}$	33.16	0.06 (0.00, 0.12)	0.035

SO_4^{2-}	5.14	0.06 (0.01, 0.12)	0.023
NO_3^-	9.08	0.00 (-0.06, 0.07)	0.946
NH_4^+	5.17	0.00 (-0.06, 0.06)	0.932
OM	8.43	0.06 (0.00, 0.11)	0.037
BC	1.51	0.09 (0.04, 0.15)	<0.001

Tables S5 Associations of $PM_{2.5}$ and its chemical components with in-hospital case fatality, using Cox regression models

Air pollutant	IQR ($\mu g/m^3$)	HR (95% CI)		
		Model 1	Model 2	Model 3
$PM_{2.5}$	33.16	1.12 (1.03, 1.23)	1.09 (0.97, 1.22)	1.08 (0.95, 1.21)
SO_4^{2-}	5.13	1.02 (0.92, 1.12)	1.01 (0.90, 1.13)	0.99 (1.00, 1.00)
NO_3^-	9.07	1.01 (0.91, 1.11)	0.91 (0.80, 1.04)	0.90 (0.78, 1.03)
NH_4^+	5.17	0.97 (0.88, 1.07)	0.89 (0.78, 1.01)	0.87 (0.76, 0.99)
OM	8.43	1.16 (1.07, 1.25)	1.12 (1.01, 1.24)	1.11 (1.00, 1.23)
BC	1.51	1.16 (1.07, 1.26)	1.12 (1.01, 1.23)	1.11 (1.00, 1.23)

Model 1: Adjusted for age, sex, ethnicity, occupation, marital status, payment methods, comorbidities and hospital level

Model 2: Adjusted for age, sex, ethnicity, occupation, marital status, payment methods, comorbidities, hospital level, temperature (ns, 3) and humidity (ns, 3)

Model 3: Adjusted for age, sex, ethnicity, occupation, marital status, payment methods, comorbidities, hospital level, temperature (ns, 5) and humidity (ns, 5)

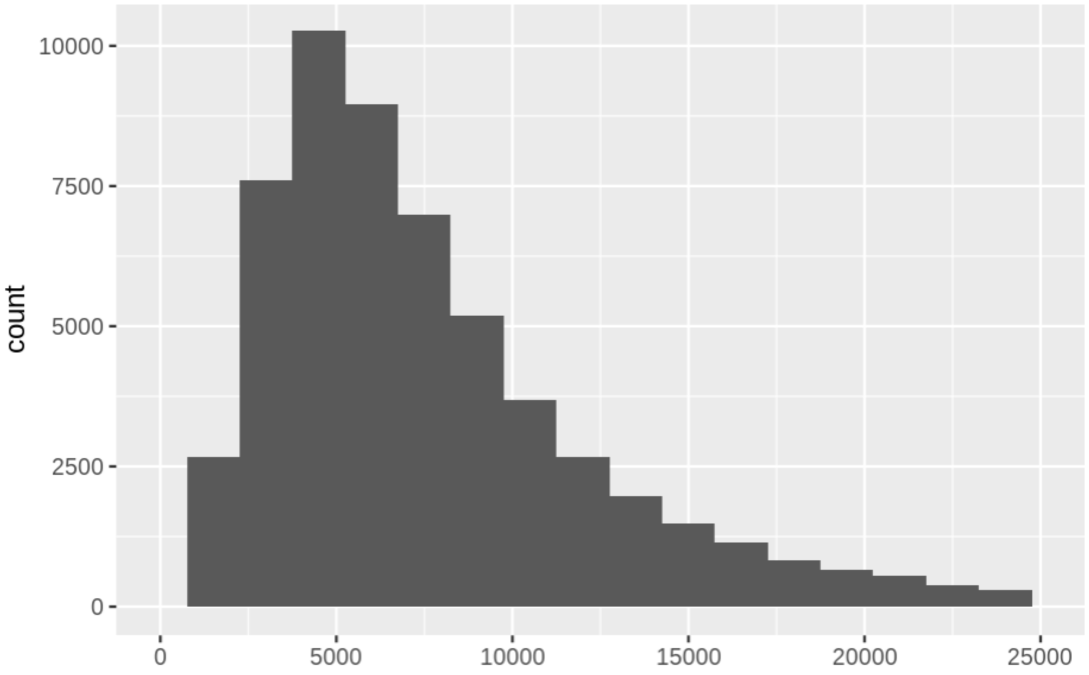

Figure S1 Distribution of hospitalization cost (RMB)

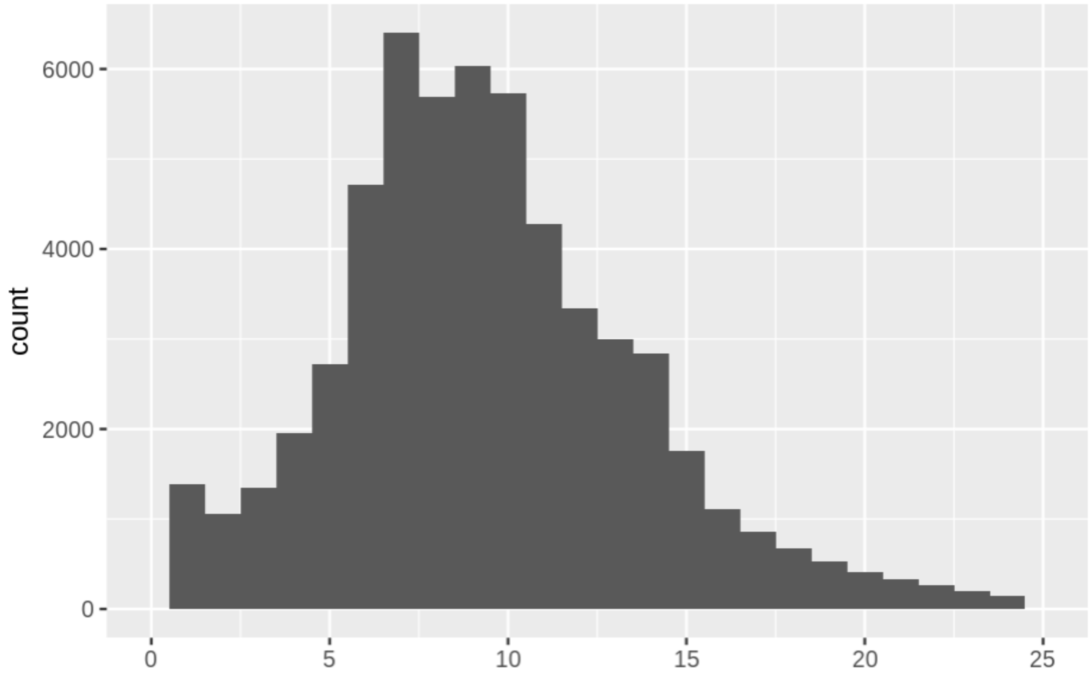

Figure S2 Distribution of length of hospital stay (LOS, day)

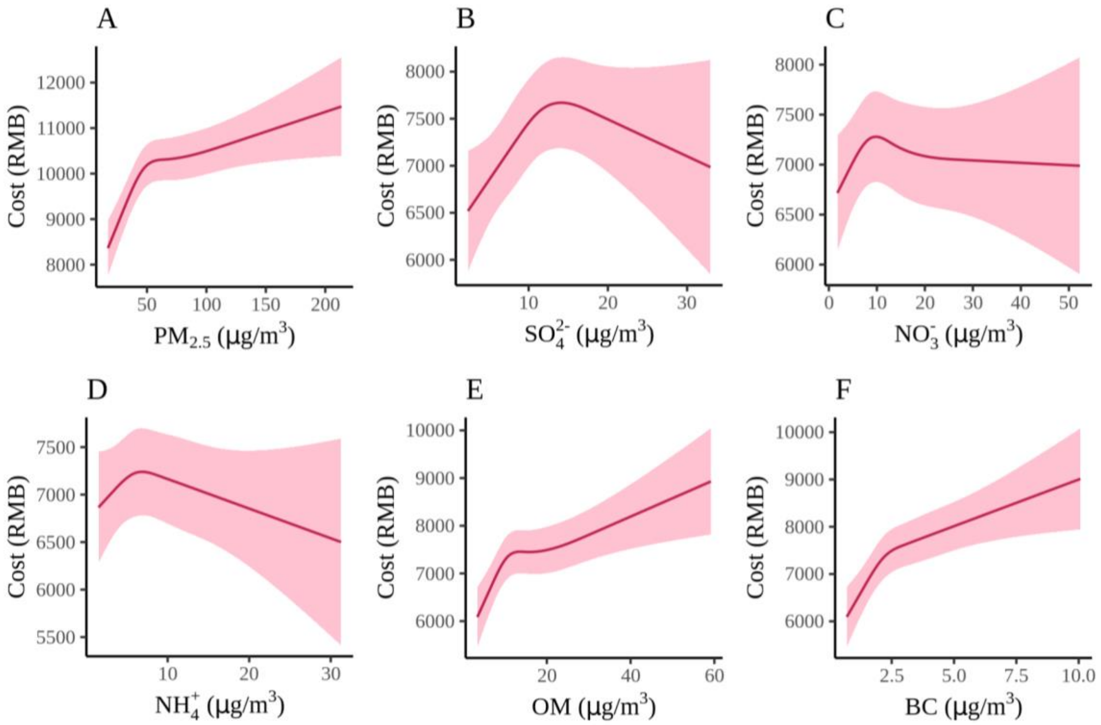

Figure S3 Exposure-response relationships of 7-day average $PM_{2.5}$ and its chemical compositions [(A) particulate matter <2.5 μm in aerodynamic diameter ($PM_{2.5}$), (B) sulphate (SO_4^{2-}), (C) nitrate (NO_3^-), (D) ammonium (NH_4^+), and (E) organic matter (OM), (F) black carbon (BC)] with HF hospitalization expenses.

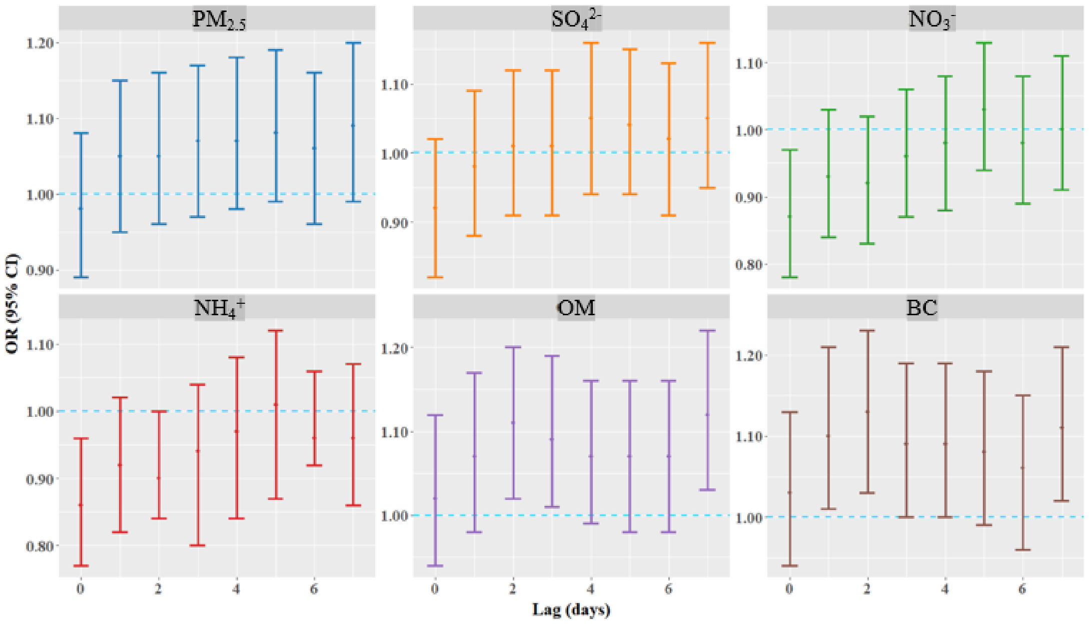

Figure S4 Associations of $PM_{2.5}$ and its chemical components with in-hospital case fatality

across the lags of 0–7 days

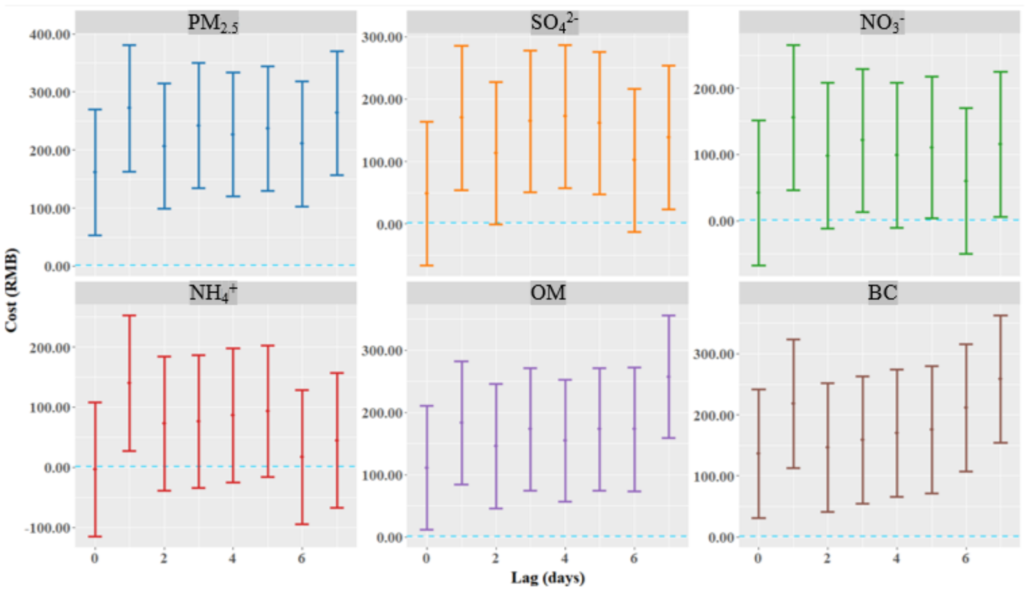

Figure S5 Associations of $PM_{2.5}$ and its chemical components with hospitalization expenses across the lags of 0–7 days

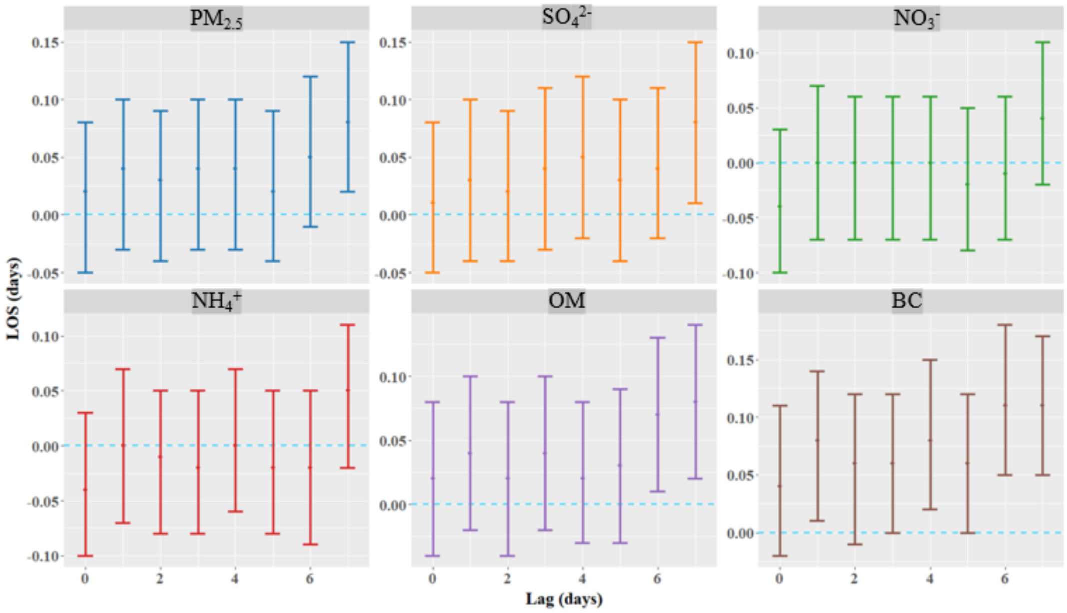

Figure S6 Associations of $PM_{2.5}$ and its chemical components with LOS across the lags of 0–7 days
